# Keeping the ageing brain wired: a role for purine signalling in regulating cellular metabolism in oligodendrocyte progenitors

**DOI:** 10.1007/s00424-021-02544-z

**Published:** 2021-03-13

**Authors:** Andrea D. Rivera, Irene Chacon-De-La-Rocha, Francesca Pieropan, Maria Papanikolau, Kasum Azim, Arthur M. Butt

**Affiliations:** 1 School of Pharmacy and Biomedical Science, University of Portsmouth, Portsmouth, UK; 2 Department of Neuroscience, Institute of Human Anatomy, University of Padua, Padua, Italy; 3 Department of Neurology, Neuroregeneration, Medical Faculty, Heinrich-Heine-University, Düsseldorf, Germany

**Keywords:** White matter, Oligodendrocyte, Oligodendrocyte precursor cell, OPC, NG2, GPR17, ATP, UDP, P2X7R, Metabolism, Myelin, Axon

## Abstract

White matter (WM) is a highly prominent feature in the human cerebrum and is comprised of bundles of myelinated axons that form the connectome of the brain. Myelin is formed by oligodendrocytes and is essential for rapid neuronal electrical communication that underlies the massive computing power of the human brain. Oligodendrocytes are generated throughout life by oligodendrocyte precursor cells (OPCs), which are identified by expression of the chondroitin sulphate proteoglycan NG2 (*Cspg4*), and are often termed NG2-glia. Adult NG2+ OPCs are slowly proliferating cells that have the stem cell–like property of self-renewal and differentiation into a pool of ‘late OPCs’ or ‘differentiation committed’ OPCs(COPs) identified by specific expression of the G-protein-coupled receptor GPR17, which are capable of differentiation into myelinating oligodendrocytes. In the adult brain, these reservoirs of OPCs and COPs ensure rapid myelination of new neuronal connections formed in response to neuronal signalling, which underpins learning and cognitive function. However, there is an age-related decline in myelination that is associated with a loss of neuronal function and cognitive decline. The underlying causes of myelin loss in ageing are manifold, but a key factor is the decay in OPC ‘stemness’ and a decline in their replenishment of COPs, which results in the ultimate failure of myelin regeneration. These changes in ageing OPCs are underpinned by dysregulation of neuronal signalling and OPC metabolic function. Here, we highlight the role of purine signalling in regulating OPC self-renewal and the potential importance of GPR17 and the P2X7 receptor subtype in age-related changes in OPC metabolism. Moreover, age is the main factor in the failure of myelination in chronic multiple sclerosis and myelin loss in Alzheimer’s disease, hence understanding the importance of purine signalling in OPC regeneration and myelination is critical for developing new strategies for promoting repair in age-dependent neuropathology.

## Introduction

**Fig. 1:**
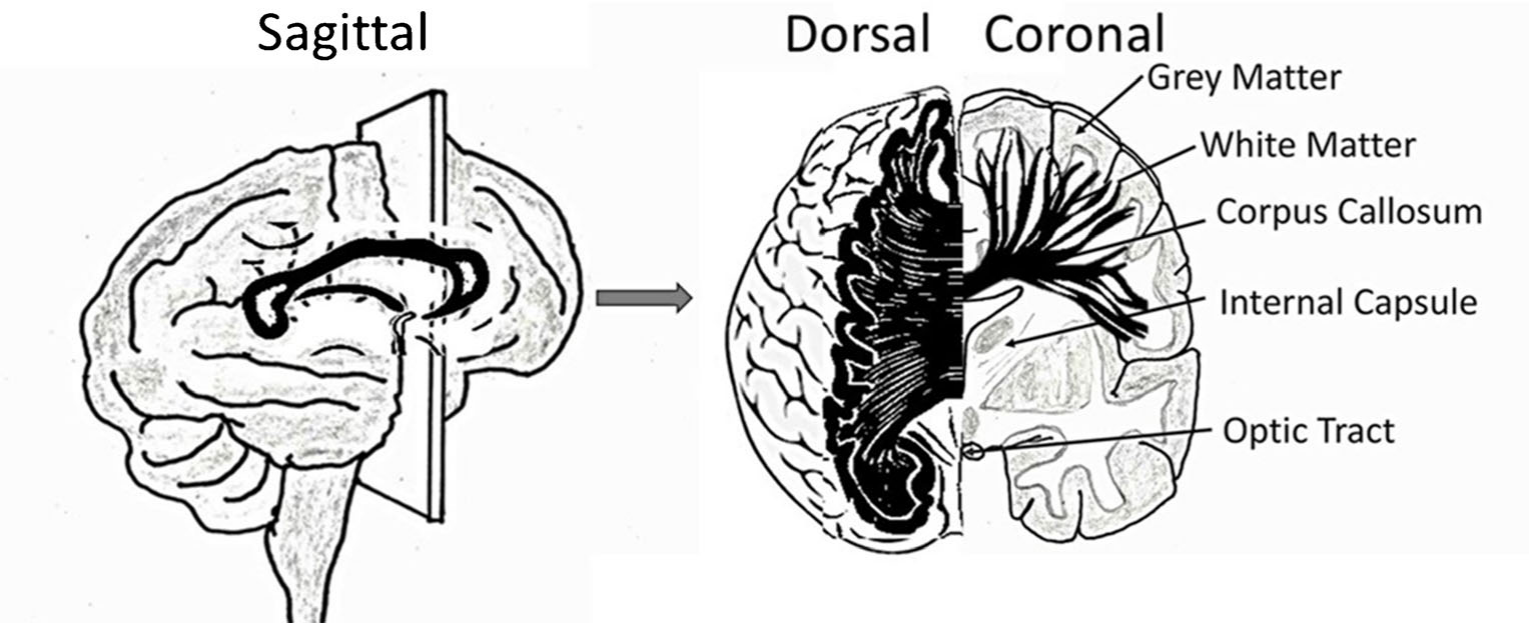
White matter is a prominent feature of human cerebral hemispheres. The white matter contains bundles of myelinated axons that interconnect neurons located in the widely dispersed grey matter areas. The corpus callosum is the largest white matter tract in the brain and interconnects the two cerebral hemispheres, shown in black in sagittal, dorsal and coronal views. Adapted from [58] and [21]

**Fig. 2:**
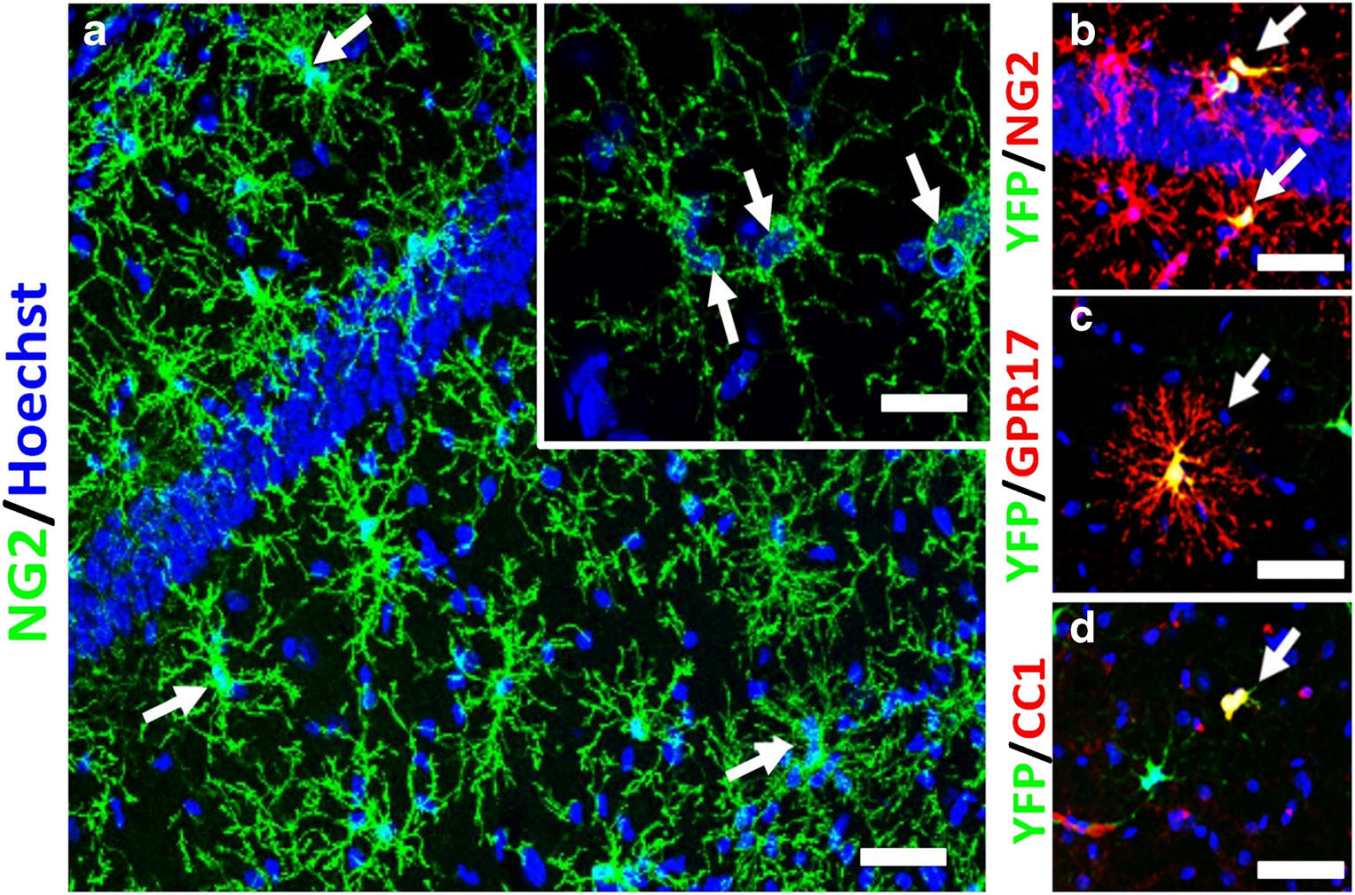
Adult NG2+/Pdgfra+ OPCs divide to undergo self-renewal and generate a reservoir of GPR17+ differentiation committed OPCs (COPs), which differentiate into CC1+ mature oligodendrocytes. **a** OPCs immunolabeled for NG2 in the hippocampus of the adult brain. White arrows show duplets of recently divided sister cells, as illustrated in the inset showing a high magnification confocal image of an OPC duplet. **b**–**d** Oligodendroglial cells in 3-month-old Pdgfra-CreER^T2^:Rosa26R-YFP mouse, 10 days following tamoxifen injection, immunolabelled for YFP to identify OPCs and their progeny (green, co-expression appears yellow), double immunofluorescence labelled for the OPC marker NG2 (B, red), GPR17 for COPs‘ (**c**, red, arrows) and CC1 for mature oligodendrocytes (**d**, red, arrows). Scale bars= 50 μm in A–D, and 10 μm in insets in **a**

White matter (WM) is a prominent feature of the human cerebral hemispheres and comprises bundles of myelinated axons that form the *connectome* of the brain (Fig. 1). Myelin is produced by oligodendrocytes and is essential for superfast communication throughout the CNS, which underlies the massive computing power of the human brain [9]. The largest WM tract in the brain is the *corpus callosum*, which is responsible for interhemispheric communication and enables higher-order functions of the cerebral cortex, including intellectual processing and behaviour [21]. Cortical function areas, such as those for language and speech, are not symmetrically represented in the two hemispheres and damage to the corpus callosum results in disconnection of the cerebral hemispheres, or ‘split brain’, when each hemisphere has separate perception, concepts and impulses to act [32]. During ageing, there is a gradual shrinkage of cerebral WM and loss of myelin, which are key factors in cognitive decline [6]. These ageing changes are accelerated in Alzheimer’s disease (AD) [55], and underlie the reduced capacity for remyelination and repair in chronic MS [67]. Furthermore, defects in callosal myelination are also features of neuropsychological disorders, including bipolar disorder, schizophrenia and autism [42]. The life-long generation of oligodendrocytes is the function of adult oligodendrocyte progenitor cells (OPCs) that are identified by expression of platelet-derived growth factor-alpha receptor (PDGFRα) and the NG2 chondroitin sulphate proteoglycan (CSPG4) (Fig. 2) [64, 81]. Significantly, myelin loss in the ageing brain is associated with diminished regenerative function of OPCs, which is the focus of this review.

## Myelination is regulated by neuronal activity and is disrupted in ageing WM

**Fig. 3:**
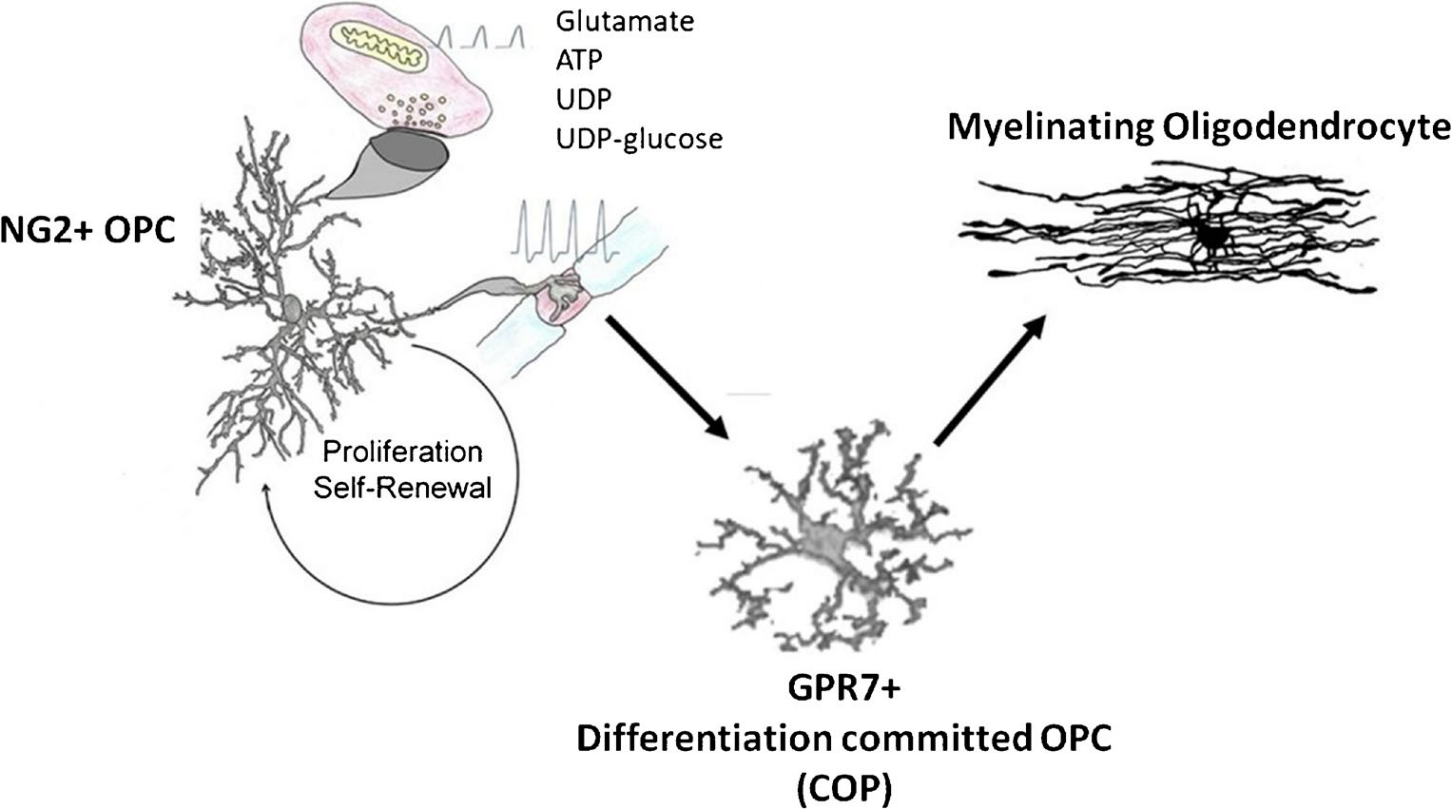
Two distinct pools of adult OPCs maintain life-long generation of myelinating oligodendrocytes. Slowly dividing NG2+ OPCs are responsible for self-renewal and maintaining a reservoir of GPR17+ COPs that are devoted to rapidly generating myelinating oligodendrocytes. Neuronal signalling involving P2X7R and GPR17, together with glutamate, noradrenaline and potassium, play an important role in regulating OPC function. There are marked decreases in both NG2+OPCs and GPR17+ COPs in the ageing brain, which results in impaired replacement of myelin lost through ageing, and is a key factor in the age-related decline in neuronal network plasticity and cognitive function

In the corpus callosum, myelination commences postnatally and is more or less complete by 10 years of age in humans (Krupa and Bekiesinska-Figatowska, 2013) and by 4 weeks in mice [64]. Nonetheless, myelination continues long into adulthood and is important for neural circuit plasticity and cognitive learning in mice and man [66]. Oligodendrocytes are generated throughout adulthood by a significant population of ‘adult OPCs’, which slowly divide to maintain their own population, termed ‘self-renewal’, and to generate newly formed oligodendrocytes [79]. A characteristic of OPCs is that they form synapses with neurons and respond to neuronal signalling [7]. Neuronal activity regulates oligodendrogenesis and myelination [35, 38, 53], which is critical for adaptive changes in learning and cognitive function [33, 69, 78]. Recent studies have determined that cortical OPCs receive extensive afferent synaptic inputs from brain-wide projection networks [54], and neurotransmission regulates both the expansion of OPCs and their differentiation into oligodendrocytes [35]. Synaptic input is important for maintaining OPC numbers [14], and age-related changes in neuronal signalling are intrinsically associated with a decline in OPC regenerative capacity [72] (Fig. 3). In this context, there are prominent roles for purinergic and glutamatergic signalling in regulating OPC proliferation and differentiation, notably via P2X7R, which are the purinergic receptor with the highest expression in OPC [43], and AMPA-type glutamate receptors [15]. Significantly, P2X7R is central to white matter pathology [28], but it is implicit that expression of P2X7R by OPCs must also have a physiological role [10], and there is evidence that P2X7R induce calcium rises in OPCs and regulate their migration, proliferation and differentiation [3, 27]. Moreover, using an NG2-DsRed mouse line to unambiguously identify NG2+ OPCs in the mouse optic nerve, we have demonstrated that OPCs respond to ATP and glutamate released by neuronal activity with increases in intracellular Ca^2+^, and showed that P2X7R and AMPAR are major contributors to such OPC functions [37]. Of course, neurotransmitters other than ATP and glutamate also regulate OPCs, including noradrenaline [12], which acts not only as a messenger, but also as a growth factor and as an inhibitor of pro-inflammatory conditions that are prominent in the aged brain [44]. Hence, reduced noradrenergic innervation in the aged brain due to the demise of the Locus coeruleus [76] may play an important role in OPC decline in ageing WM, either directly or via altered astrocyte function [58]. Furthermore, OPCs sense potassium released during neuronal activity through the potassium channels they express, notably Kir4.1, as well as sensing metabolites, including L-lactate, which represent further modes of OPC signal integration [12, 58]. Overall, OPC self-renewal is at least partly dependent on neuronal activity and age-related dysregulation of neurotransmission is a potential causative factor in the loss of OPCs and myelin [13, 73].

## The adult brain contains distinct pools of NG2+ and GPR17+ OPCs that are altered in ageing

**Fig. 4:**
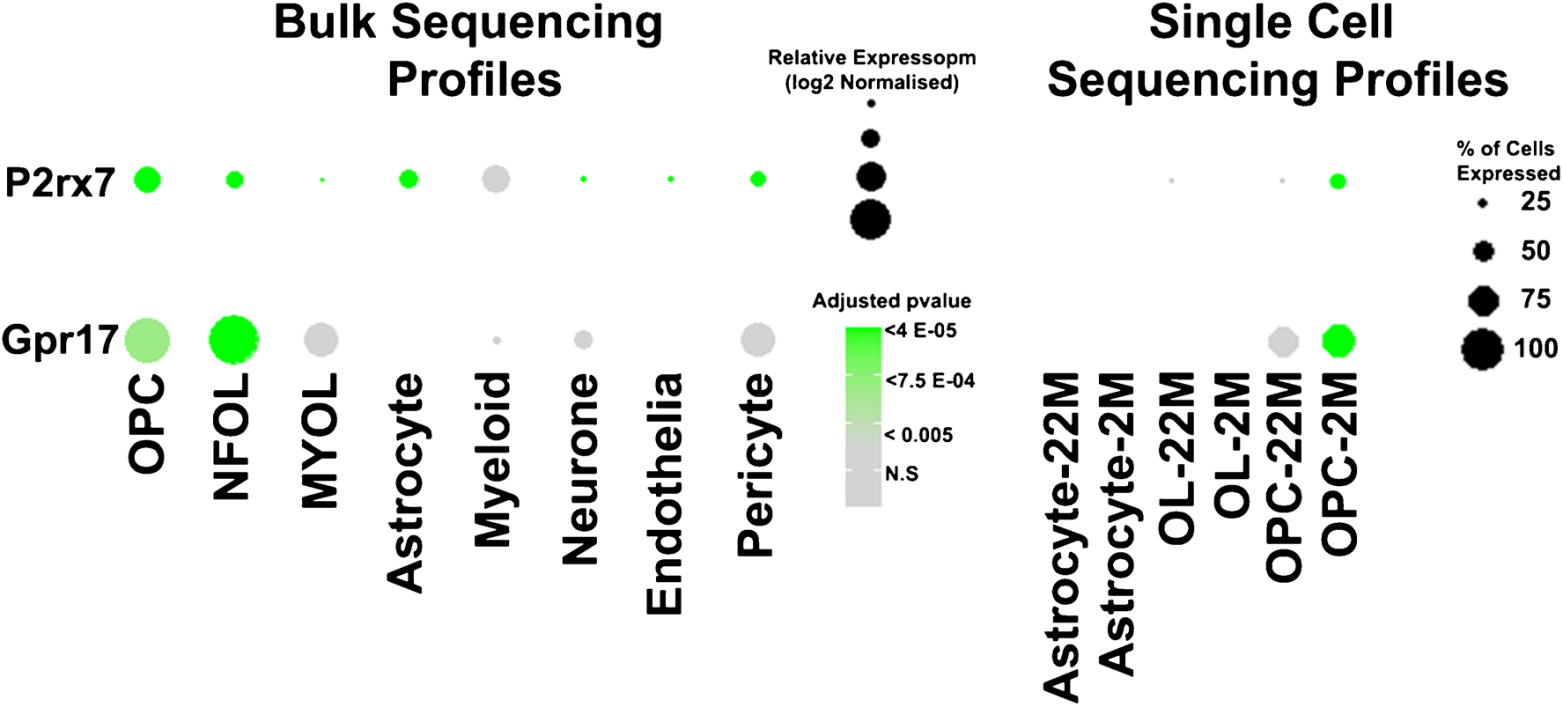
Specific expression of GPR17 and P2RX7 in mouse oligodendroglial cells. Transcript signals values of mouse brain cells from publically available datasets of bulk sequencing [80] and single-cell sequencing [40] were analysed using the DeSeq2, Seurat and ggplot2 packages in RStudio, following standard published procedures [59, 63]. Bulk sequencing profiles indicate highest expression of *P2rx7* corresponds to *Gpr17* in OPCs and COPs, compared to other cells. This was confirmed in single-cell sequencing profiles, which indicate a marked decrease in expression levels of both *P2rx7* and *Gpr17* in aged OPCs (22 months of age, 22 M) compared to adult OPCs (2 months of age, 2 M)

**Fig. 5:**
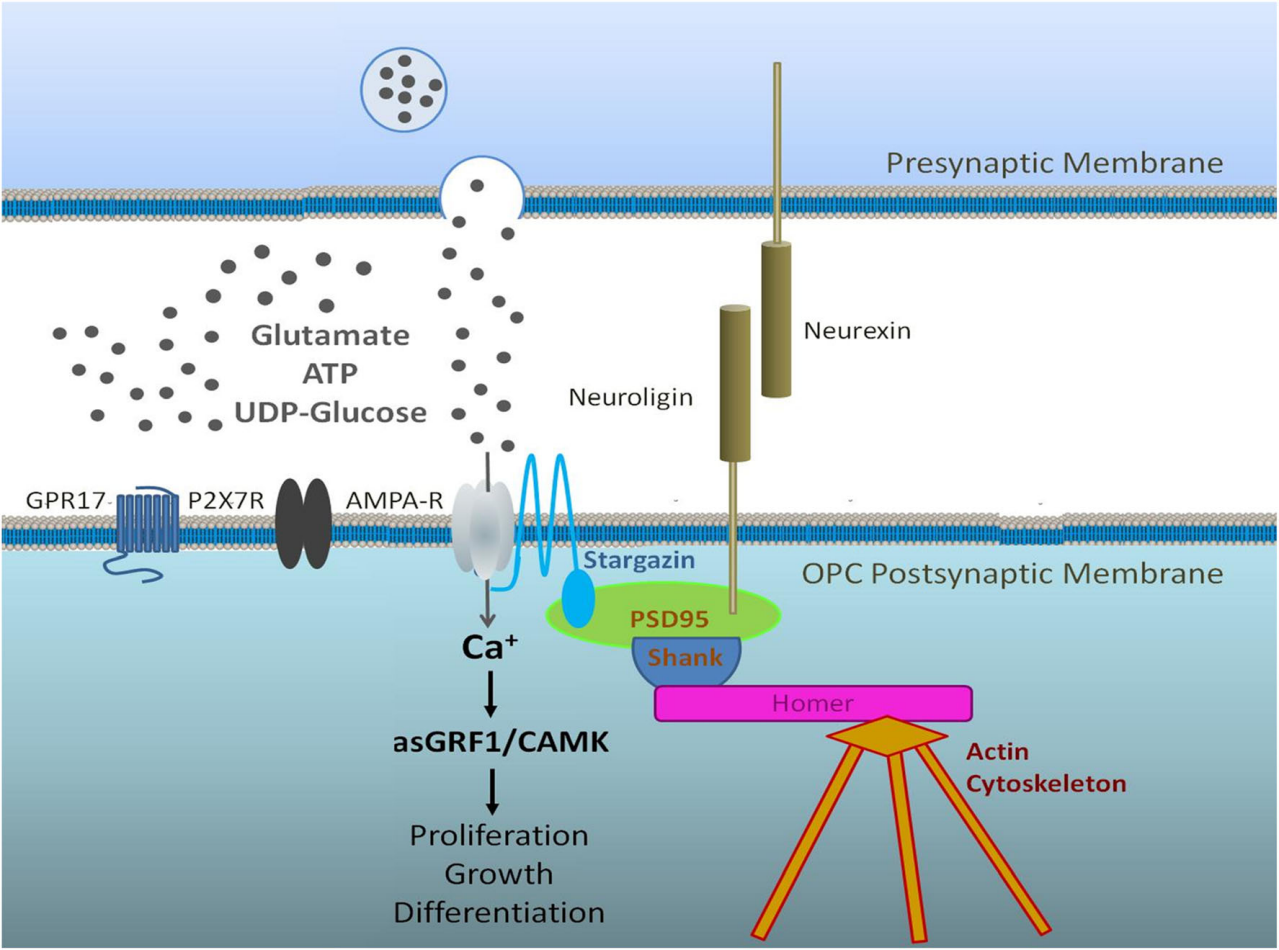
Prominent roles for purinergic and glutamatergic signalling in regulating OPC proliferation and differentiation.AMPA-type glutamate receptors and P2X7R subtype of ATP receptors are highly expressed by OPCs, which are charecterised by expression of the NG2 CSPG and GPR17. Potential interactions between GPR17, P2X7R and the targeting of AMPA receptors to the OPC cell membrane are implicated in  age-related dysregulation of OPCs

During development, OPCs arise from focal sources to migrate throughout the CNS, where they proliferate and differentiate into oligodendrocytes, under the control of multifarious intrinsic and extrinsic factors [26]. Although adult OPCs are generally treated as a single uniform population, it is evident they are a heterogeneous population and that not all OPCs are directly involved in the generation of myelinating oligodendrocytes [11]. Prior to differentiating into mature myelinating oligodendrocytes, NG2+ OPCs pass through a distinct differentiation phase characterised by expression of the G-protein-coupled receptor subtype GPR17 [30] (Fig. 4). Single-cell RNAseq has identified multiple cell subpopulations belonging to the oligodendrocyte lineage and GPR17 expression was identified in clusters that can be collectively defined as ‘differentiation committed OPC’ (COPs) [49]. In support of this, analysis of OPC heterogeneity in zebrafish spinal cord revealed that GPR17 is differentially expressed in a subset of OPCs that generate differentiated oligodendrocytes, while it is virtually absent in a subgroup of OPCs that seem to be more involved in synaptic signalling [48]. Adult OPCs can be broadly subdivided into two functionally distinct pools of slowly dividing NG2+ OPCs that have the stem cell–like property of self-renewal [12], and GPR17+ COPs, that are normally quiescent and differentiate into myelinating oligodendrocytes when needed [18, 75]. Thus, the function of NG2+ OPCs is self-renewal and replenishment of GPR17+ COPs, which serve as ‘reservoir’ of cells devoted to rapidly generating myelinating oligodendrocytes [45]. It is significant, therefore, that we have recently demonstrated a marked decrease in both NG2+OPCs and GPR17+ COPs in the ageing brain [63], caused by reduced self-renewal of OPCs [56], together with their diminished replenishment of the reservoir of GPR17+ COPs [63]. The dysregulation of OPCs and COPs results in impaired replacement of myelin lost through ageing [56, 63], which is a key factor in the age-related decline in neuronal network plasticity and cognitive function [6], and for myelin loss in AD and the failure of remyelination in chronic MS [55, 67]. Transcriptomic studies are beginning to unravel the signalling pathways and biological processes that are altered in aged OPCs, and notable amongst these are cell metabolism and synaptic signalling [20, 63]. Our meta-analysis of the stage-specific transcriptional signatures of ageing cortical OPC identified novel interactions between *Gpr17* and *Cacng4* (Stargazin) [63], which targets AMPA receptors to the OPC cell membrane [82], to regulate OPC proliferation, differentiation and myelination [15]. Furthermore, P2X7R have also been shown to regulate AMPAR trafficking and enhance glutamatergic synaptic signalling [60]. Transcriptomics evidence has indicated both AMPAR and P2X7R are disrupted in ageing white matter [72]. Moreover, GPR17 and P2X7R are both important regulators of cellular metabolism [17, 61], which is central to age-related dysregulation of OPCs [56]. Thus, the collective disruption of GPR17 and P2X7R could result in severe disruption of aged OPCs, as both genes are detected at very low expression compared to young OPCs (Fig. 5), in addition to other factors, such as reduced noradrenergic signalling and metabolic support by astrocytes [58, 74, 76].

## Roles for P2X7R and GPR17 in OPC ageing and cellular metabolism

P2X7R are usually considered cytotoxic receptors, butalso have an important physiological function as sensors of cellular metabolic state and, on activation, can regulate cell metabolism [22]. Furthermore, although it is generally held that P2X7R are activated only at pathologically high concentrations of extracellular ATP, there is evidence that ATP is released physiologically in the CNS during neuronal activity at high enough concentrations to activate P2X7R, as a neurotransmitter in its own right, or as a co-transmitter with glutamate or other neurotransmitters [1]. A recent study measured ATP changes in response to neuronal activity in the cerebral cortex of living mice and demonstrated an ATP wave that propagated at the speed of ~2 mm/min, comparable to the rate of neuronal propagation, with a precipitous rise of ATP at the wave front that occurred across a broad area of the brain [41]. In addition, we have shown an equivalent rise in ATP in response to neuronal activity that is propagated by ATP release from astrocytes in WM of the mouse optic nerve [36], at elevated levels sufficient to activate P2X7R on OPCs [37]. Furthermore, it is now evident that ATP is continuously released in the brain and extracellular levels are altered in response to metabolic demand [25, 47, 51], linked to reciprocal changes in the levels of phosphorylated AMP-activated protein kinase (P-AMPK), well known for its role in cellular energy sensing and regulation [25]. Controlled activation ofP2X7R supports mitochondrial ATP synthesis in multiple ways, including facilitating glucose uptake by regulating glucose transporter expression and function [4], and stimulation ofP2X7R has been shown to enhance energy metabolism in mice [34]. In addition, circadian regulation of extracellular ATP levels suggests that ATP may be an important circadian output in thesuprachiasmatic nucleus and other brain regions [77]. Similarly, physiological ligands for GPR17 include UDP and UDP-glucose[16], which are important factors in glycogenesis [29], and may be released from neurons and astrocytes to activate receptors on OPCs [24]. Importantly, oligodendroglial GPR17 has been shown to regulate whole-body metabolism and food intake by modulating hypothalamic neuronal activity [57, 61, 70]. In this context, P2X7R and GPR17 act as bioenergetics sensors and provide mechanisms by which OPCs regulate cellular metabolism and survival. Moreover, evidence that activation of GPR17 by uracil nucleotides is reversed by some purinergic antagonists [30] raises the possibility of interactions between GPR17 and P2X7R. It is significant, therefore, that GPR17 and P2X7R regulate whole-body metabolism and are implicated in type-2 diabetes [17, 61] and regulate mTOR and AMPK [8, 62], which also contribute to type-2 diabetes [39], since dysregulation of these pathways are central to age-related changes in OPCs and targeting these pathways rejuvenates ageing OPC stemness [56, 63]. Moreover, activation of P2X7R modulates GSK3β and intracellular glycogen stores [23], and we have demonstrated that inhibition of GSK3β profoundly promotes oligodendrogenesis and rejuvenates the regenerative capacity of OPCs [5]. In this respect, it is worth noting that astrocytes are the primary store of glycogen in the brain, which is a source of metabolic support for OPCs [58]. Moreover, activation of GPR17 in OPCs decreases intracellular levels of cAMP [68], which is an important regulator of glycogenolysis, and the decline in excitation-energy coupling is likely to be an important factor in the ageing brain [74].

## Oligodendrocytes provide metabolic support for axons

In addition to myelination, oligodendrocytes provide metabolic support for axons, possibly in the form of glucose [52], but mainly by delivering lactate to axons, which they release through MCT1 into the periaxonal space, from where it is taken up by axons via MCT2 [31, 46]. Metabolic support is coupled to axonal activity, which stimulates oligodendroglial expression of the glucose transporter GLUT1 and glucose uptake, which is metabolised to lactate and released to axons [65]. The physiological importance of oligodendrocyte-axon metabolic support is critical under conditions of glucose deprivation [71]. Interestingly, downregulation of GPR17 in oligodendroglia enhanced glycolysis and lactate production, which then activated neurons in the hypothalamus [57], suggesting that the loss of GPR17 during the maturation of myelinating oligodendrocytes would increase their capacity for metabolic support of axons. As noted above, oligodendroglial P2X7R are activated by ATP released during neuronal activity [36, 37], and extracellular ATP levels increase under metabolic stress [51]. Furthermore, evidence that activation of P2X7R regulates glycolysis and facilitates glucose uptake via increased GLUT1 expression and function [4] provides a physiological function for oligodendroglial P2X7R in supporting axonal metabolism and integrity, as shown for glutamate and NMDAR [65]. Hence, in addition to resulting in dysregulation of OPC cellular metabolism, age-related changes in oligodendroglial P2X7R and GPR17 may have adverse effects on axonal metabolism, in particular under times of metabolic stress, which is a hallmark of brain ageing [50], and is postulated to play a key role in neuronal demise in AD [19] and MS [2].

## Summary and conclusions

In summary, WM shrinkage and myelin loss in the ageing brain underpin a decline in neuronal plasticity and cognitive function. A key factor in the loss of myelin is the age-related decay in OPC regenerative capacity, which is associated with dysregulation of cellular metobilism. Notably, OPCs express GPR17 and P2X7R, which are key regulators of OPC differentiation and can be considered bioenergetics sensors that regulate cellular metabolism to meet changes in energy demands. Overall, evidence for dysregulation of GPR17 and P2X7R in ageing supports key roles for these receptors in the age-related loss of OPC stemness and their regeneration of myelinating oligodendrocytes. Furthermore, such changes in P2X7R and GPR17 would have adverse effects on axonal trophic support and is likely to contribute to neurodegenerative changes in ageing WM, both through disruption of their metabolic roles in oligodendroglial glycolysis and their importance in regulating oligodendrocyte regeneration and myelination. Thus, P2X7R and GPR17 are potential therapeutic targets for rejuvenating OPCs and promoting myelin repair and neuroprotection in age-dependent neuropathology, including MS and AD.
